# Overall survival of metastatic melanoma patients treated with HD IL-2 followed by immune checkpoint blockade of the CTLA-4 or the PD-1 pathways: analysis of data on the current use of HD IL-2

**DOI:** 10.1186/2051-1426-3-S2-P359

**Published:** 2015-11-04

**Authors:** Michael KK Wong, Michael A Morse, David F McDermott, Joseph I Clark, Howard L Kaufman, Gregory A Daniels, Jessica C Perritt, Hong Hua, Sandra Aung

**Affiliations:** 1 University of Southern California, Los Angeles, Los Angeles, CA, USA; 2 Duke University Medical Center, Durham, NC, USA; 3 The Cytokine Working Group; Division of Hematology/Oncology, Beth Israel Deaconess Medical Center, Boston, MA, USA; 4 Loyola University Medical Center, Division of Hematology Oncology, Maywood, IL, USA; 5 Rutgers Cancer Center Institute of New Jersey, New Brunswick, NJ, USA; 6 Moores Cancer Center, University of California San Diego, La Jolla, CA, USA; 7 Prometheus Laboratories Inc., San Diego, CA, USA

## Background

HD IL-2 was FDA approved for advanced melanoma, but the data supporting its use dates to the 1990's. The PROCLAIM^SM^ registry (http://www.proclaimregistry.com) is the largest collection of IL-2 treated patients in the US and provides real-time insights into patient survival and outcomes. Previously, we reported a median overall survival (mOS) of 20 months with a median follow-up of 37 months in metastatic melanoma (mM) patients treated with high dose IL-2 (HD IL-2) between 2007 and 2012 from a retrospective cohort. These findings led to the hypothesis that improved mOS may have been a result of subsequent salvage therapies, including checkpoint inhibitors.

## Methods

Patients must have received at least one dose of HD IL-2 for this analysis. Those that received checkpoint therapy prior to HD IL-2 were excluded. Statistics and survival analysis on prospectively entered patients (N=236) were performed on datasets as of March 16^th^, 2015.

## Results

The median overall survival (mOS) for the 236 patients was 18.4 months with a median follow-up of 21.7 months. Patients were stratified into three groups; HD IL-2 only (n=123), HD IL-2 followed by ipilimumab (IL-2→ipi, n=78), and HD IL-2 followed by PD-1 inhibitors (IL-2→aPD-1, n=35). The majority of patients (22 of 35) in the IL-2→aPD-1 group had progressive disease before receiving subsequent treatment with anti-PD-1/PD-L1-containing regimens. Patients in the HD IL-2 only, IL-2→ipi, and IL-2→aPD-1 groups achieved a mOS of 14, 15.8, and 28.7 months, respectively. The estimated 12-month survival rates were 57%, 64%, and 97%, respectively. There were 10/78 (13%) and 2/35 (5.7%) post therapy treatment-related incidences of autoimmune events in the IL-2→ipi and IL-2→aPD-1 groups, respectively. No treatment related deaths were reported.

## Conclusions

This is the first report of clinical data relating to HD IL-2 use followed by checkpoint blockade of the PD-1 pathway. Treatment with anti-PD-1 after initial therapy with HD IL-2 had significantly prolonged survival compared to patients treated with ipilimumab. Moreover, improved survival was not observed in patients treated with follow-on ipilimumab compared to patients treated only with HD IL-2. Anti-PD-1 therapy after HD IL-2, appears to be safe and therapeutically active. These data support the concept of investigating IL-2 therapy in combination or sequence with newly developed immune checkpoint inhibitors.

